# Contribution of personalized Cyclin D1 genotype to triple negative breast cancer risk

**DOI:** 10.7603/s40681-014-0003-4

**Published:** 2014-08-27

**Authors:** Liang-Chih Liu, Chen-Hsien Su, Hwei-Chung Wang, Wen-Shin Chang, Chia-Wen Tsai, Ming-Chei Maa, Chang-Hai Tsai, Fuu-Jen Tsai, Da-Tian Bau

**Affiliations:** 1 Terry Fox Cancer Research Laboratory, China Medical University Hospital, 2 Yuh-Der Road, 404 Taichung, Taiwan; 2 Graduate Institute of Basic Medical Science, China Medical University, Taichung, Taiwan; 3 Department of Medical Research, China Medical University Hospital, Taichung, Taiwan; 4 Graduate Institute of Clinical Medical Science, China Medical University, Taichung, Taiwan

**Keywords:** *Cyclin D1*, Genotype, Triple negative breast cancer

## Abstract

Aim: Cell cycle regulator *cyclin D1 (CCND1)* is a pivotal regulator for G1/S phase transition, playing a critical part in initiation of carcinogenesis. Triple negative breast cancer comprises a very heterogeneous group of cancer cells, but little is known about what is wrong in the genome of these patients. This study investigated contribution of *CCND1* genotype to individual triple negative breast cancer susceptibility.

Materials: In all, 2464 native Taiwan subjects consist of 1232 breast cancer cases and 1232 controls were enrolled in a hospital-based, case-control study. *CCND1* A870G (rs9344) genotyping was analyzed by polymerase chain reaction-restriction fragment length polymorphism (PCR-RFLP). Risk-stratified analyses correlated genotype and age-related characteristics of breast cancer subgroups.

Results: No significant difference was found between patient and control groups in distribution of genotypic and allelic frequencies in *CCND1* genotype, yet *CCND1* A870G (rs9344) GG genotype was far less prevalent in breast cancer patients younger than 55 years (OR=0.62, 95%CI=0.43–0.89, *P*=0.0362), with first menarche earlier than 12.2 years (OR=0.61, 95% CI=0.42–0.87, P=0.0241), with menopause earlier than 49.0 years (OR=0.57, 95%CI=0.39–0.82, *P*=0.0093), or showing triple-negative breast cancer (OR=0.28, 95%CI=0.13–0.62, *P*=0.0006). Such valuable findings suggest *CCND1* A870G (rs9344) as a predictive marker for triple negative breast cancer in Taiwanese women; the authors sincerely hope these help us fight the toughest subtype in clinical management.

## 1. Introduction

Breast cancer is one of the most common worldwide malignancies in women today; its morbidity and mortality have not decreased with development of anticancer drugs [1]. Breast cancer in Asia displays lower incidence than in Western populations, but is still the leading cancer among Asian women and an issue of extraordinary public health concern. Asian breast cancer is characterized by early tumor onset, showing a relatively younger median age at diagnosis. In Taiwan, breast cancer ranks second among cancers, noted for high incidence, high mortality, and early onset [2, 3]. Most women are exposed to well-known environmental risk factors for cancer, but only a portion of exposed individuals develop breast cancer, suggesting a wide variation in individual susceptibility.

Cyclin D1 (CCND1) plays a critical role in controlling G1/S phase transition of the cell cycle [4], which accomplishes this gate-keeping role by forming a complex with its partners CDK 4 or CDK6 [4-5]. Some reports demonstrate it as involved in some types of tumor growth in a CDK-independent pattern [6-7]. Dysregulation of CCND1 is commonly observed in human cancer, with overexpression of it frequently cited as a potential biomarker [8-10]. However, underlying mechanisms of CCND1 overexpression and its connection to breast cancer progression are poorly understood. Terry Fox Cancer Research Lab in China Medical University previously found that *CCND1* genotypes positively associated with other types of cancer in Taiwan [11-15]. We currently take interest not only in revealing the contribution of genotypes to breast cancer, but to its toughest subtype in clinical treatment: triple negative breast cancer.

This study’s genotyping work ascertained correlation between *CCND1* A870G (rs9344) polymorphism and breast cancer risk in Taiwanese women. Additional analyses evaluated the contribution of this SNP to breast cancer patients with specific clinicopathological features, such as those of triple negative breast cancer.

## 2. Materials and methods

### 2.1. Study population

A total of 1232 patients diagnosed with breast cancer were recruited at the outpatient clinics of general surgery at China Medical University Hospital in Taichung, Taiwan. Clinical characteristics of patients (including histological details) were all defined by expert surgeons. Slides were reviewed and scored by two independent pathologists. For ER, PR, and p53 immunoassaying, nuclear stain in 10% of neoplastic cells served as positive cutoff, Ki67-labelling index of >30% considered positive. HER-2/neu results were derived according to the package insert and guidelines of the American Society of Clinical Oncology and College of American Pathologists [16]. All patients voluntarily participated, completing self-administered questionnaires and supplying peripheral blood samples. An equal number of age-matched non-breast cancer healthy volunteers as controls were selected after initial random sampling from the hospital’s Health Examination Cohort. Exclusion criteria of the control group included previous malignancy, metastasized cancer from other or unknown origin, and any familial or genetic disease. Both groups completed a short questionnaire that included habits. Our study was approved by the Institutional Review Board of China Medical University Hospital (DMR96-IRB-240), written-informed consent obtained from all participants.

### 2.2. Genotyping conditions

Genomic DNA was prepared from peripheral blood leukocytes using a QIAamp Blood Mini Kit (Blossom, Taipei, Taiwan) and genotyping processes performed as in our prior studies [11-15]. Briefly, primers used for *CCND1* A870G were: forward 5’-GTG AAG TTC ATT TCC AAT CCG C-3’, and reverse 5’-GGG ACA TCA CCC TCA CTT AC-3’. Polymerase chain reaction (PCR) cycling conditions were: one cycle at 94°C for 5 min; 35 cycles of 94°C for 30 s, 55°C for 30 s, and 72°C for 30 s, and a final extension at 72°C for 10 min.

### 2.3. RFLP conditions

After PCR procedure for *CCND1* A870G genotyping, resultant 167 bp PCR product was mixed with 2 U *Nci* I and incubated for 3 h at 37　C. The G form PCR products could be further digested, while A form could not. Two fragments 145 bp and 22 bp were present if the product was digestible G form. Then 10 　l of product was loaded into a 3% agarose gel containing ethidium bromide for electrophoresis, genotype analysis performed by two researchers independently and blindly. Ten percent of the samples were randomly selected for direct sequencing, results entirely concordant.

### 2.4. Statistical analyses

To ensure controls representative of general population while precluding genotypic error, genotype frequency deviation of *CCND1* single nucleotide polymorphisms in controls from those expected under Hardy-Weinberg equilibrium was assessed using the goodness-of-fit test. Pearson’s Chi-square or Fisher’s exact test (when expected number in any cell was less than five) compared distribution of *CCND1* genotypes between groups, statistical *P*-value less than 0.05 recognized as significant.

## 3. Results

A total of 1232 patients diagnosed with breast cancer and an equal number of matched controls were enrolled, as compared and summarized in Table 1. Ages of patients and controls were well matched, as were age at menarche, age when bearing first child (*P*>0.05) (Table 1). As for individual behavior, tobacco smoking and alcoholism both emerged as risk factors for breast cancer in this population (*P*<0.05) (Table 1).

Table 2 plots frequencies of genotypes and alleles of CCND1 A870G in breast cancer and control groups. First, results of genotyping analysis revealed distribution of *CCND1* A870G genotype do not significantly differ between patients and controls (*P*=0.1949) (Table 2). Odds ratios of AG and GG were 0.95 and 0.80 (95% CI= 0.79-1.15 and 0.62-1.03) compared to AA wild-type genotype. Second, we performed dominant and recessive comparison to find odds ratios of GG versus AA+AG and AG+GG versus AA were 0.82 and 0.92 (95%CI=0.66-1.03 and 0.77-1.10, P=0.0931 and 0.3793), respectively. Last, there was no significant difference between breast cancer and controls in distribution of allelic frequency (OR=0.92, 95%CI=0.82-1.03, *P*=0.1442): i.e., G allele (AG and GG) meant a slightly but not statistically protective effect against breast cancer compared to AA wild genotype (Table 2).

We took interest in association of clinicopathologic traits with *CCND1* A870G genotypes. Given diverse mechanisms of carcinogenesis in distinct subtypes of breast cancer, we analyzed linkage among *CCND1* A870G genotypes with age-related and clinicopathologic characteristics of breast cancer patients (Tables 3-4). Data showed GG genotype at *CCND1* A870G less prevalent in breast cancer patients younger than 55 years (OR=0.62, 95%CI=0.43–0.89, *P*=0.0362), with first menarche earlier than 12.2 years (OR=0.61, 95% CI=0.42–0.87, *P*=0.0241), with menopause earlier than 49.0 years (OR=0.57, 95%CI=0.39–0.82, *P*=0.0093), or with triple-negative breast cancer (OR=0.28, 95%CI=0.13–0.62, P=0.0006) (Tables 3-4). Different genotype distribution among breast cancer patients stratified by other factors, including first full pregnant (Table 3) and Ki67 status (Table 4), was not statistically significant.

**Table 1.** Distribution of demographic and life-style of breast cancer patients and matched controls

Characteristic	Controls (n = 1232)			Patients (n = 1232)			*P*-value
	n	%	Mean (SD)	n	%	Mean (SD)	
Age (years)							
< 40	359	29.1%		362	29.4%		0.89^a^
40-55	558	45.3%		547	44.4%		
> 55	315	25.6%		323	26.2%		
Age at menarche (years)			12.4 (0.7)			12.1 (0.6)	0.79^b^
Age at birth of first child (years)			29.4 (1.2)			29.8 (1.4)	0.63^b^
Age at menopause (years)			48.8 (1.8)			49.3 (2.0)	0.59^b^
Site							
Unilateral				1198	97.2%		
Bilateral				34	2.8%		
Family History							
First degree (Mother, sister and daughter)				55	4.5%		
Second degree				6	0.5%		
No history				1171	95%		
Habit							
Cigarette smokers	86	7.0%		170	13.8%		<0.0001^a^
Alcohol drinkers	91	7.4%		162	13.1%		<0.0001^a^

Statistic results based on ^a^ Chi-square or ^b^ unpaired *Student’s t*-test.

**Table 2.** Intergroup distribution of *CCND1* A870G (rs9344) genetic and allelic frequencies

A870G (rs9344)	Controls	%	Patients	%	OR (95% CI)^a^	*P*-value^b^
Genetic frequency						
AA	303	24.6%	323	26.2%	1.00 (Reference)	0.1949
AG	725	58.8%	736	59.7%	0.95 (0.79-1.15)	
GG	204	16.6%	173	14.1%	0.80 (0.62-1.03)	
Carrier comparison						
AA+AG	1028	83.4%	1059	85.9%	1.00 (Reference)	0.0931
GG	204	16.6%	173	14.1%	0.82 (0.66-1.03)	
AA	303	24.6%	323	26.2%	1.00 (Reference)	0.3793
AG+GG	929	75.4%	909	73.8%	0.92 (0.77-1.10)	
Allele frequency						
Allele A	1331	54.0%	1382	56.1%	1.00 (Reference)	0.1442
Allele G	1133	46.0%	1082	43.9%	0.92 (0.82-1.03)	

^a^ OR: odds ratio, CI: confidence interval; ^b^ Based on Chi-square test

**Table 3.** Association of *CCND1* A870G genotypes with age-related related demographic characteristics

Characteristics	*CCND1 A870G*			
	Controls N (%)	Cases n (%)	P-value^a^	Crude^b^ OR (95% CI)^c^
**Onset age**				
<55.0 years			**0.0362** ^*^	
AA	146 (23.06)	169 (27.57)		1.00 (Ref^d^)
AG	377 (59.56)	365 (59.54)		0.84 (0.64-1.09)
GG	110 (17.38)	79 (12.89)		**0.62** **(** **0.43-0.89** **)** ^*^
AG+GG	487 (76.94)	444 (72.43)		0.79 (0.61-1.02)
≧55.0 years			0.8040	
AA	157 (26.21)	154 (24.88)		1.00 (Ref^d^)
AG	348 (58.10)	371 (59.94)		1.09 (0.83-1.42)
GG	94 (15.69)	94 (15.18)		1.02 (0.71-1.46)
AG+GG	442 (73.79)	465 (75.12)		1.07 (0.83-1.39)
**Age at menarche**				
<12.2 years			**0.0241** ^*^	
AA	146 (23.70)	171 (27.85)		1.00 (Ref^d^)
AG	360 (58.44)	365 (59.45)		0.87 (0.66-1.13)
GG	110 (17.86)	78 (12.70)		**0.61 (0.42-0.87)** ^*^
AG+GG	470 (76.30)	443 (72.15)		0.80 (0.62-1.04)
≧ 12.2 years			0.9362	
AA	157 (25.49)	152 (24.60)		1.00 (Ref^1^)
AG	365 (59.25)	371 (60.03)		1.05 (0.80-1.37)
GG	94 (15.26)	95 (15.37)		1.04 (0.73-1.50)
AG+GG	459 (74.51)	466 (75.40)		1.05 (0.81-1.36)
**Age at first birth of child**				
< 29.6 years			0.4570	
AA	148 (24.03)	161 (26.26)		1.00 (Ref^1^)
AG	365 (59.25)	363 (59.22)		0.91 (0.70-1.19)
GG	103 (16.72)	89 (14.52)		0.79 (0.55-1.14)
AG+GG	468 (75.97)	452 (73.74)		0.89 (0.69-1.15)
≧ 29.6 years			0.3791	
AA	155 (25.16)	162 (26.17)		1.00 (Ref^d^)
AG	360 (58.44)	373 (60.26)		0.99 (0.76-1.29)
GG	101 (16.40)	84 (13.57)		0.80 (0.55-1.14)
AG+GG	461 (74.84)	457 (73.83)		0.95 (0.73-1.22)
**Age at menopause**				
< 49.0 years			**0.0093** ^*^	
AA	144 (23.38)	177 (28.64)		1.00 (Ref^d^)
AG	364 (59.09)	366 (59.22)		0.82 (0.63-1.06)
GG	108 (17.53)	75 (12.14)		**0.57 (0.39-0.82** ^*^
AG+GG	472 (76.62)	441 (71.36)		**0.76 (0.59-0.98)** ^*^
≧49.0 years			0.7110	
AA	159 (25.81)	146 (23.78)		1.00 (Ref^d^)
AG	361 (58.60)	370 (60.26)		1.12 (0.85-1.46)
GG	96 (15.59)	98 (15.96)		1.11 (0.78-1.59)
AG+GG	457 (74.19)	468 (76.22)		1.12 (0.86-1.45)

^a^ Based on Chi-square.

^b^ Difference in the trend in statistical significance before any adjustment for individual habits such as smoking (pack-years).

^c^ OR, odds ratio; CI, confidence interval.

^d^ Ref., reference.

^*^ Statistical significant

**Table 4.** Association of *CCND1* A870G genotypes with breast cancer risk stratified by clinicopathologic characteristics compared with non-cancer healthy controls

Character	Genotype, number (%)^a^			OR (95 %CI)^b^	P-value^c^
	AA	AG	GG		
Control	303 (24.6)	725 (58.8)	204 (16.6)	1.00 (Ref^1^)	
Triple-negative status					
No	159 (28.8)	316 (57.1)	78 (14.1)	0.73(0.53-1.01)	0.1228
Yes	42 (40.4)	54 (51.9)	8 (7.7)	**0.28 (0.13-0.62)** ^*^	**0.0006** ^*^
Ki67 status					
Negative	76 (27.4)	156 (56.3)	45 (16.2)	0.88(0.58-1.32)	0.6099
Positive	90 (26.6)	193 (57.1)	55 (16.2)	0.91 (0.62-1.33)	0.7447

^a^ Triple-negative and Ki67 status data were available for 657 and 615 patients, respectively, all data given as number of patients (%) unless otherwise noted.

^b^ OR, odds ratio; CI, confidence interval.

^c^ Based on Chi-square.

^d^ Ref., reference.

^*^ Statistical significant

## 4. Discussion

For years, Terry Fox Cancer Research Lab in China Medical University has keeping on the anticancer task via the translational circle from genomic biomarker revealing, anticancer drug discovery, cell and animal model establishment for drug efficacy and genotype-phenotype correlation investigation, and clinical personalized application. In this hospital-based case-control study, our team has genotyped a famous SNP *CCND1* A870G studying its association with Taiwanese breast cancer risk in central Taiwan. With a collection of samples from a quite large population, we have found that the GG genotype in *CCND1* A870G plays a protective role for triple-negative breast cancer, and in early onset (< 55 years), early menarche (<12.2 years) and premenopausal (<49 years) Taiwanese women.

As a first step, we performed routine genotype work, but results showed *CCND1* A870G genotype not linked with breast cancer susceptibility. Since we have almost collected the largest breast cancer population in Taiwan (1232 cases and age-matched controls), strategy of investigating more subjects is less urgent. Estrogen exposure is widely viewed as closely related to breast cancer risk, with age undeniably the strongest demographic risk factor for most malignancies (75% occur in patients older than 55 years) [17]. With adequate sample size, we confidently rated the contribution of this SNP to breast cancer patients with specific clinicopathological features by stratification analysis. The estrogen- and age-related factors included onset age, age at menarche, age at first birth of child, and age at menopause (Table 3). Likewise, we wished to evaluate contribution of this SNP to triple negative breast cancer. This study identified 104 breast cancer patients with triple negative breast cancer. So-named because of its negative expression of ER, PR, and HER-2/neu [18], it is characterized by aggressiveness and higher rates of recurrence and metastasis. Interestingly, existing targeted therapies effective against other subtypes of breast cancer were ineffective in dealing with triple negative. It typically occurs in young patients, whose disease is associated with variations of BRCA1 and other genes: e.g., *hOGG1, EGFR2* [16, 19, 20]. Cyclin D1 (coded by *CCND1*) plays first gatekeeper in the cell cycle, whereas copy number alterations of *CCND1* were reported as differentially more frequent in triple negative breast cancer samples than those in other breast cancers [21]. Still, no report investigates association of SNPs on *CCND1* with triple negative breast cancer risk. Our results proved genotype of *CCND1* A870G not correlated with breast cancer risk as in other malignancies [11-15]; more promising, stratified analysis showed GG genotype of *CCND1* A870G playing a protective role for triple-negative breast cancer (Table 4), as with early onset (<55 years), early menarche (<12.2 years) and/or premenopausal (<49 years) Taiwanese women (Table 3). It is also found that Ki67 status, reported as a potential indicator to triple negative breast cancer [22], were not associated with *CCND1* A870G genotype (Table 4).

Recent years have seen rapidly accumulated information on cancer genotyping as a great boost for translational medicine and personalized therapy. We still have a long way to go to make history in this field. The first successful step seemed fulfilled by cooperation between local clinicians and basic scientists. Since heredity plays a key role in cancer susceptibility, we must pay more attention to genetic conservation and independence of Taiwan from Western countries, respecting profound ethnic differences while pursuing globalization. Translational studies in Taiwan, participation of experts in nutrition and care-taking, together with cooperation of patients and relatives, warrant bolstering and encouragement. Our study highlights GG genotype of *CCND1* A870G playing a protective role in triple-negative breast cancer, as well as in early onset, early menarche and premenopausal Taiwanese women. We sincerely hope each successive piece of our work expedites personalized therapy and medication, plus the war against cancer, especially in our beloved Taiwan.

## Acknowledgements

This work was supported by China Medical University Hospital (grant number: DMR-103-019) and the Terry Fox Cancer Research Foundation. The authors deeply appreciate help from Tsai-Ping Ho, Chieh-Lun Hsiao, Lin-Lin Hou, Chao-Yi Lai, Chia-En Miao, Tzu-Chia Wang, Yun-Ru Syu and Tissue Bank.
